# The effect of fish oil supplementation on resistance training-induced adaptations

**DOI:** 10.1080/15502783.2023.2174704

**Published:** 2023-02-23

**Authors:** Jeffery L. Heileson, Steven B. Machek, Dillon R. Harris, Sara Tomek, Leticia C. de Souza, Adam J. Kieffer, Nicholas D. Barringer, Andrew Gallucci, Jeffrey S. Forsse, LesLee K. Funderburk

**Affiliations:** a Department of Health, Human Performance, and Recreation, Baylor University, Waco, TX, USA; b Nutrition Services Division, Walter Reed National Military Medical Center, Bethesda, MD, USA; c Kinesiology Department, College of Health Sciences and Human Services, California State University - Monterey Bay, Seaside, CA, USA; d Department of Educational Psychology, Baylor University, Waco, TX, USA; e Department of Nutrition, U.S. Military-Baylor University Graduate Program in Nutrition, Fort Sam Houston, TX, USA; f Department of Human Sciences and Design, Baylor University, Waco, TX, USA

**Keywords:** Hypertrophy, strength, body composition, eicosapentaenoic acid, docosahexaenoic acid

## Abstract

**Background:**

Resistance exercise training (RET) is a common and well-established method to induce hypertrophy and improvement in strength. Interestingly, fish oil supplementation (FOS) may augment RET-induced adaptations. However, few studies have been conducted on young, healthy adults.

**Methods:**

A randomized, placebo-controlled design was used to determine the effect of FOS, a concentrated source of eicosapentaenoic acid (EPA) and docosahexaenoic acid (DHA), compared to placebo (PL) on RET-induced adaptations following a 10-week RET program (3 days·week^−1^). Body composition was measured by dual-energy x-ray absorptiometry (LBM, fat mass [FM], percent body fat [%BF]) and strength was measured by 1-repetition maximum barbell back squat (1RM_SQT_) and bench press (1RM_BP_) at PRE (week 0) and POST (10 weeks). Supplement compliance was assessed via self-report and bottle collection every two weeks and via fatty acid dried blood spot collection at PRE and POST. An *a priori* α-level of 0.05 was used to determine statistical significance and Cohen’s *d* was used to quantify effect sizes (ES).

**Results:**

Twenty-one of 28 male and female participants (FOS, *n* = 10 [4 withdrawals]; PL, *n* = 11 [3 withdrawals]) completed the 10-week progressive RET program and PRE/POST measurements. After 10-weeks, blood EPA+DHA substantially increased in the FOS group (+109.7%, *p*< .001) and did not change in the PL group (+1.3%, *p* = .938). Similar between-group changes in LBM (FOS: +3.4%, PL: +2.4%, *p* = .457), FM (FOS: −5.2%, PL: 0.0%, *p* = .092), and %BF (FOS: −5.9%, PL: −2.5%, *p* = .136) were observed, although, the between-group ES was considered large for FM (*d* = 0.84). Absolute and relative (kg·kg [body mass]^−1^) 1RM_BP_ was significantly higher in the FOS group compared to PL (FOS: +17.7% vs. PL: +9.7%, *p* = .047; FOS: +17.6% vs. PL: +7.3%, *p* = .011; respectively), whereas absolute 1RM_SQT_ was similar between conditions (FOS: +28.8% vs. PL: +20.5%, *p* = .191). Relative 1RM_SQT_ was higher in the FOS group (FOS: +29.3% vs. PL: +17.9%, *p* = .045).

**Conclusions:**

When combined with RET, FOS improves absolute and relative 1RM upper-body and relative 1RM lower-body strength to a greater extent than that observed in the PL group of young, recreationally trained adults.

## 1. Background

The preservation and promotion of skeletal muscle mass and strength is critical for physical performance and healthy physiology throughout the lifespan [1]. Resistance exercise training (RET) may be one of the best- and well-established strategies to influence these parameters [2]. Muscle protein synthesis (MPS), an important determinant of muscle mass and commensurate strength enhancements, is stimulated by RET [3]. The most common and widely recognized nutritional strategy to augment RET-induced adaptations, including MPS, is the intake of dietary protein (1.2-1.6 g·kg^−1^), especially the provision of essential amino acids [4]. Recently, long-chain omega-3 polyunsaturated fatty acids (LC n-3 PUFA), primarily eicosapentaenoic acid (EPA), and docosahexaenoic acid (DHA), have been investigated for their roles in MPS and skeletal muscle health [5].

The incorporation of EPA and DHA into skeletal muscle phospholipid has been shown to enhance both nutrient and mechanically sensitive anabolic signaling proteins known to regulate MPS [6]. Moreover, fish oil supplementation (FOS), a concentrated source of EPA and DHA, augments the anabolic response to nutrient stimuli in healthy young and middle-aged men and women through the activation of the mTOR-p70s6k signaling pathway leading to a ~ 50% increase in MPS [7]. A study in resistance-trained young men demonstrated that FOS augmented the anabolic response elicited by protein feeding alone and with the addition of RET relative to placebo (PL), as indicated by a ~ 30% and 35% increase in MPS, respectively, in the absence of a concomitant increase in kinase signaling activity [8]. Moreover, these findings have corroborated similar alterations in protein signaling following skeletal muscle EPA and/or DHA incorporation [9,10]. As such, it appears that FOS may attenuate signaling cascades without compromising functional outcomes such as muscular hypertrophy and strength. Unlike targeted pharmaceutical interventions, LC n-3 PUFAs may act via several other mechanisms that may influence RET-induced adaptations such as muscle quality associated factors such as muscle fiber type transition or enhanced neuromuscular recruitment; muscle protein breakdown; improved insulin signaling; enhanced cell membrane fluidity; and modulation of inflammatory cytokines [5,11–13].

While plausible mechanisms exist, it is unclear if FOS influences functional skeletal muscle outcomes such as the promotion of hypertrophy, strength, and fat mass (FM) reduction in young adults following a RET program [14,15]. As noted by Anthony et al. [16] and James et al. [17], n-3 PUFA research is plagued with methodological flaws that may render some interpretations of primary outcomes tenuous at best. Since the influence of EPA and DHA on physiology is mediated by incorporation into tissue phospholipid membranes, the aforementioned authors proposed the use of a membrane-centric hypothesis to resolve these issues in n-3 PUFA research [16]. For example, two studies investigating the effects of RET and n-3 PUFA supplementation on skeletal muscle outcomes in young adults did not measure membrane nor blood LC n-3 PUFA status, likely employing both suboptimal EPA and DHA dosing (<1 g·d^−1^) and duration (4-8 weeks) protocols to meaningfully influence skeletal muscle incorporation [18,19]. Furthermore, as stated by Rossato et al. [15] and others [14], it is apparent from the lack of available data that concurrent RET and FOS trials should be undertaken, appropriately, to determine if LC n-3 PUFAs augment skeletal muscle functional outcomes in young adults.

Therefore, the aim of this study was to determine if there is an augmented response of FOS (3.85 g∙d^−1^ EPA+DHA), compared to PL, on body composition (LBM, FM, %BF) and 1-repetition maximum (1RM) strength during a 10-week RET program in young adults. Based on the current literature, we hypothesized that an adequate dose of FOS alongside RET, would facilitate increased LBM and strength, as well as a reduction in FM to a greater magnitude than RET plus PL. If so, targeted RET and FOS interventions can be developed.

## 2. Methods

### Study design

**Figure 1.**
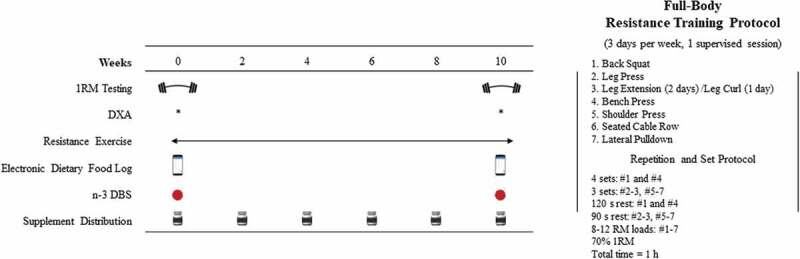
Schematic Overview of the Study Design and Resistance Exercise Training Protocol.

A randomized, single-blind, parallel-group design was used to examine the effects of FOS compared to placebo (PL) on body composition and strength during a 10-week RET program in young adults. Regarding blinding, one of the outcome assessors was aware of the allocations; however, all outcomes were conducted with other investigators present (see the *Strength Testing* section). A schematic overview of the study design is depicted in Figure 1.

### Subjects

**Figure 2.**
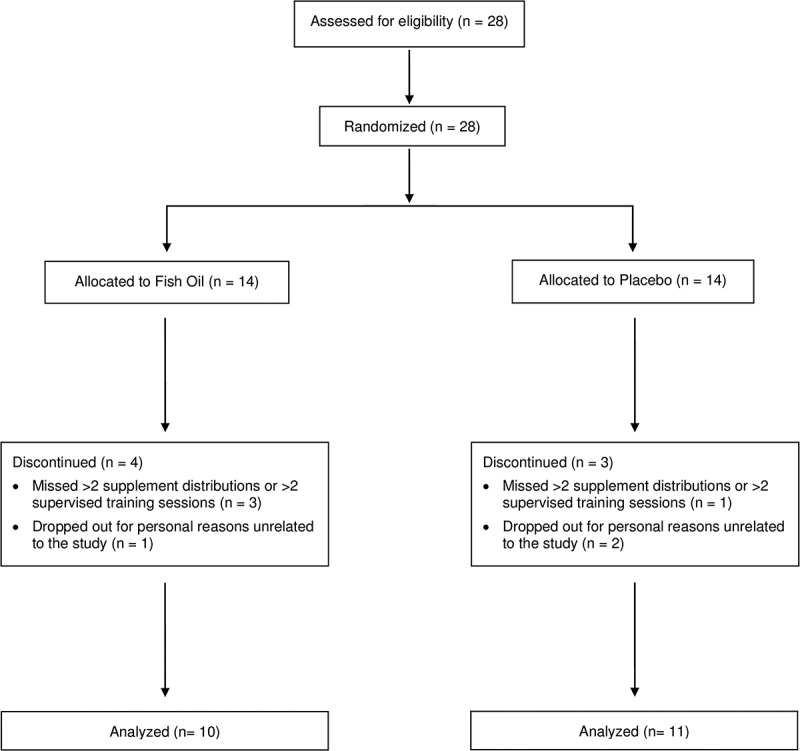
CONSORT Participant Flow Diagram.

Twenty-eight young male (n = 12) and female (n = 16) adults were recruited from the local central Texas area and university population for this study. All participants met the following criteria: 1) between 18 and 40 years, 2) free from neuromuscular/musculoskeletal disorders and known chronic diseases (heart disease, type-2 diabetes mellitus, etc.), 3) did not regularly consume ergogenic or fish oil supplements within 6 months of starting the study, 4) consumed <2 servings of fatty fish per week, 5) did not take anabolic steroids or selective androgen receptor modulators, 6) reported both being recreationally trained (defined as RET twice per week for at least 6 months) and familiar (i.e. conducted both movements weekly for at least 6 months) with the barbell back squat and barbell bench press, and 7) have a body fat percentage (%BF) ≤26 in males and ≤36 in females. After briefing all study details, eligible subjects signed university-approved written consent forms. This study was approved by the Institutional Review Board for Human Subjects at Baylor University (#1630023-6). Of the initial 28 participants recruited, 7 withdrew from the study. Figure 2 outlines subject recruitment, randomization, and reasons for drop out.

Since there is little evidence to suggest sex differences in the anabolic response from RET with similar training statues [20], we opted to include male and female subjects. While hormonal variation does appear to uniquely impact women physiologically, there is no clear evidence that the menstrual cycle or oral contraceptive use significantly influences physical performance [21,22]. Furthermore, recent data suggests that RET-induced changes in hypertrophy and strength are minimally influenced by sex [23]. While some evidence suggests a differential response after FOS based on sex in older adults [24], this finding was not replicated in a recent trial [11] and has not been observed in young adults. Based on a recent dose-response study, we also anticipated similar changes in the red blood cell EPA+DHA following omega-3 supplementation, regardless of sex or age [25].

### Supplementation protocol

Groups were supplemented with FO (4.5 g∙d^−1^ [2.275 g∙d^−1^ EPA + 1.575 g∙d^−1^ DHA], 7 capsules; Nordic Naturals, ProOmega, Watsonville, CA, USA) or PL (safflower oil, 4.5 g∙d^−1^, 5 capsules; NOW, Bloomingdale, IL, USA) for 10-weeks. Based on recent data on skeletal muscle phospholipid incorporation of EPA+DHA following supplementation [6,26], our FOS dose needed to be >3 g∙d^−1^. To account for possible missed doses, we opted to provide a slightly higher dose (3.85 g∙d^−1^ EPA+DHA). Supplements were distributed every two weeks to encourage compliance. Adherence was confirmed by verbal confirmation and upon visual inspection of the bottles by the same laboratory technician. Additionally, blood fatty acid status was determined via fatty acid dried blood spot (DBS) as muscle and blood EPA+DHA content are highly correlated [6]. The supplements were packaged in similar bottles and the capsules were similar in size and shape in an attempt to blind the subject to the allocation. To assess blinding, at the end of the study, subjects were asked to guess which group they were in.

### Resistance training procedures

As illustrated in Figure 1, subjects conducted a partially supervised 10-week full-body RET protocol in three nonconsecutive sessions/days per week (two unsupervised and one supervised) consisting of seven exercises per session of 3-4 sets of 8-12 repetitions with 90-120 s rest intervals. See Figure 1 for the complete RET protocol. Briefly, the following exercises were performed in order: barbell back squat, leg press, leg extension/leg curl, barbell bench press, shoulder press, seated cable row, and wide-grip lat pulldown. Similar exercise regimes have been used previously to study various hypertrophy and strength outcomes [27,28]. One RET session per week was supervised by trained exercise physiologist and research personnel, while the other two RET sessions were completed by the participant for a total of 10 supervised sessions and 20 unsupervised sessions over 10-weeks. Subjects were prohibited from performing additional RET or high-intensity anaerobic training until the completion of the study.

Initial training loads were selected based on 70% of the subject’s baseline 1RMs. The load was adjusted for all exercises based on the subject’s ability to reach momentary concentric failure between 8-12 repetitions. The load was decreased at the next training session if the subject completed less than eight repetitions on the final set or increased if the subject was able to complete all 12 repetitions on the final set. Load adjustments were approximately 5-10% for each exercise.

### Measurements

*Body Composition*. Total body mass (kg) and height (cm) were determined using a standard scale with a stadiometer (Seca 703, Hamburg, Germany). At PRE and POST, body composition (%BF, FM, and LBM) was obtained under laboratory conditions (e.g. fasted, voided bladder, and same time of day) using dual-energy x-ray absorptiometry (DXA, Horizon DXA™, Hologic®, Bedford, MA). The same technician performed each DXA to minimize variability. Based on previous studies in our lab, the accuracy of the DXA is ± 2 to 3.7% as compared to hydrodensitometry [29].

*Strength Testing*. Lower- and upper-body strengths were assessed by 1RM testing via the back squat (1RM_SQT_) and bench press (1RM_BP_) exercises, respectively. Subjects conducted a 5-minute bike warm-up then a self-directed dynamic warm-up for an additional 5-minutes that included one set of 10 repetitions with an unloaded 20.4 kg barbell. Subjects then completed a standardized warm-up protocol used previously in our lab consisting of 10 repetitions at approximately 50% 1RM, 5 repetitions at 70% 1RM, 3 repetitions at 80% 1RM, and 1 repetition at 90% 1RM [30]. All warm-ups were followed by 2-minute rest intervals. Subjects then performed sets of one repetition with increasing weight for 1RM determination. During the testing phases, 3-minute rest intervals were employed and all 1RM determinations were made within five attempts. For the 1RM_SQT_, subjects were required to reach parallel, in which the top of the thigh is discernably parallel to the floor, for a repetition to be considered successful. For the 1RM_BP_, the lift was deemed successful if the subject kept five points of contact (bench: head, upper back, buttocks; ground: both feet), touched the barbell to their chest (no pause), and executed a full lock-out. While we do not have lab-specific reliability measures for the 1RM_BP_ and 1RM_SQT_, at least two study personnel were available for spotting and standards verification. All strength testing sessions were supervised, and standards additionally verified by the lead technician and one additional technician – all National Strength and Conditioning Association Certified Strength and Conditioning Specialists. The average PRE and POST 1RM_SQT_ and 1RM_BP_ were reported as absolute and relative to body weight (1RM [kg]∙body mass [kg]^−1^) values.

*Volume Load*. Volume load data, calculated as sets x reps x load, were obtained from bench press and back squat for each session. Combined volume load and volume load data for back squat and bench press separately from each week were averaged across 3-RET sessions and used for data analysis.

### Dietary records

Subjects were required to submit three-day food logs (two weekdays, one weekend day) before and after the RET intervention using the MyFitnessPal mobile or desktop application (MyFitnessPal; San Francisco, CA, USA). Additionally, LC n-3 PUFA intake was assessed using a food frequency questionnaire (FFQ) that has been validated against whole blood EPA and DHA [31]. While the subject diets were not standardized, they were asked to keep their dietary habits as consistent as possible. Additionally, subjects were asked to consume at least 1.0 g∙kg^−1^ per day of protein and given gram-specific targets per day by a registered dietitian. Macronutrients (kcals, protein, carbohydrate, fat) and n-3 fatty acids intake (EPA and DHA) were averaged both over the three-day tracking period and per group for analysis by the same registered dietitian. Lastly, macronutrient data were normalized to body mass (kg) for further analysis.

### Fatty acid dried blood spot

Fatty acid dried blood spot (DBS) was obtained to track supplementation compliance and ensure adequate LC n-3 PUFA membrane incorporation. A drop of blood was collected from each participant via finger stick on filter paper pre-treated with a preservation solution (Fatty Acid Preservative Solution, FAPS™) and allowed to dry at room temperature for ~15 minutes. At the conclusion of the study, the DBS were shipped overnight on dry ice to OmegaQuant (Sioux Falls, SD, USA) for fatty acid analysis. Based on their standard laboratory protocol, fatty acids were identified by comparison with a standard mixture of fatty acids characteristic of RBC (GLC OQ-A, NuCheck Prep, Elysian, MN, USA) and used to construct individual fatty acid calibration curves. Fatty acid composition was expressed as a percent of total identified fatty acids. PRE and POST values of EPA, DHA, and the omega-3 index (O3i) were reported. The O3i is defined as the sum of EPA and DHA adjusted by a regression equation (*r* = 0.96) to predict the RBC O3i.

### Statistical analyses

All statistical analyses were performed using IBM SPSS version 28 (Armonk, NY, USA). Data were tested for normality and homogeneity using the Shapiro-Wilks and Levene’s tests, respectively. Baseline characteristics were analyzed using an independent samples *t*-test. The sample size for this project was 26. This sample size was justified by *a priori* power analysis in G*power using a target effect size (ES) of f = 0.35, alpha of 0.05 and power of 0.80, which determined that 20 subjects were required for participation with an additional number of participants recruited to account for possible attrition. Of note, similar RET investigations with a nutritional intervention used identical per group sample sizes (n = 8-11) [18,19], even with cohorts including males and females [32]. The primary outcome data (body composition [LBM, FM, %BF], and strength [1RM_SQT_ and 1RM_BP_]) were analyzed using an ANCOVA on the change scores with baseline values as the covariate. All other data with timepoints (PRE/POST: 1RM strength relative to body weight [kg], fatty acids [O3i, EPA, DHA], dietary variables [kcals, protein, carbohydrate, fats, EPA, and DHA], weeks 1-10: volume load) were analyzed using a two-way repeated measures ANOVA (group x time). If the assumption of sphericity (Mauchly’s test) was violated, the Greenhouse-Geisser correction was used. If significant interaction effects were present, pairwise comparison analyses were used with a Bonferroni adjustment for alpha inflation. Significance was set a priori at *p* < .05. ES values are reported as Cohen’s *d* to infer the between-group magnitude of differences in change scores. ES values were classified according to Cohen [33] as trivial, < 0.2; small, 0.2 – 0.49; moderate, 0.5 – 0.79; and large, ≥ 0.8. All data presented as mean ± SD, unless otherwise stated.

## 3. Results

Seven subjects dropped out during the study, resulting in a total of 21 subjects (FOS group, n = 10 [M: 5, F: 5); PL group, n = 11 [M: 5, F: 6]). Reasons for dropouts are noted in the participant flow diagram (Figure 2). The FO and PL groups had similar (*p* > .05) baseline characteristics (Table 1).

**Table 1.** Baseline participant characteristics.

	Fish Oil (n = 10; 5 men, 5 women)	Placebo (n = 11; 5 men, 6 women)	*p*-value
Age (*y*)	28.0 (7.4)	30.5 (5.7)	.403
Height (*cm*)	169.7 (9.6)	171.8 (8.9)	.679
Weight (*kg*)	75.1 (16.0)	79.0 (16.0)	.906
BMI (*kg*∙*m^−2^*)	25.8 (3.5)	26.6 (4.3)	.496
Body Fat (%)	23.9 (6.9)	24.9 (8.0)	.766
Training Age (*y*)	1.8 (1.1)	2.0 (1.0)	.652
Omega-3 Index (%)	4.9 (1.3)	4.3 (0.9)	.209

Data are mean (SD). Omega-3 Index = %EPA + %DHA in red blood cells

### Compliance

*Supplement*. Self-reported supplement compliance was 95.2% for all participants. There was no difference in supplement compliance between groups (FOS: 94.6%, PL: 95.8%, *F* (1,19) = 0.331, *p* = .572). Fifty-seven percent of subjects (12 of 21; FOS: 6 of 10, PL: 6 of 11) were unable to ascertain their allocated group. Only two subjects in the FOS group reported experiencing ‘fishy burps’. No other symptoms or adverse effects were reported.

*RET Protocol*. Overall attendance for those who completed the study was similar between groups (supervised: *F* (1,19) = 0.022, *p* = .883; unsupervised; *F* (1,19) = 0.1118, *p* = .734). Participants in the PL and FOS groups had an 94.6% and 95.0% attendance for the RET supervised sessions, respectively, and a self-reported unsupervised session attendance of 95.0% and 94.0%, respectively.

**Figure 3.**
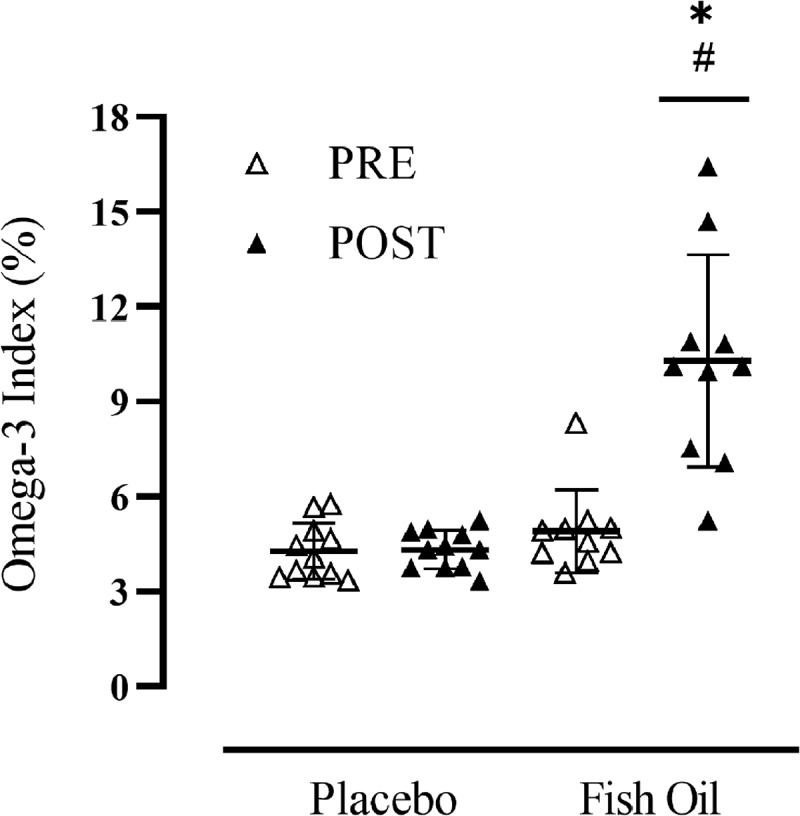
Participant Omega-3 Index Before (PRE) and After (POST) 10-Weeks of Supplementation. Black line with whiskers indicates mean ± SD.*significantly different than PRE (*p* < .001), #significant difference between groups (*p* < .001).

*Fatty Acid Dried Blood Spot*. The baseline average O3i for all subjects was 4.58% ± 1.12 (FOS: 4.9% ± 1.3, PL: 4.3% ± 0.9). There were no baseline group differences in the O3i (*F* (1,19) = 1.688, *p* = .209) or whole blood values of EPA (*F* (1,19) = 0.309, *p* = .585) and DHA (*F* (1,19) = 1.829, *p* = .192). As noted in Figure 3, the O3i did not change in the PL group (1.3%, *p* = .938) and significantly increased from PRE to POST in the FOS group (109.7%, *p* < .001). Similarly, whole blood EPA and DHA did not significantly change in the PL group (14.7%, *p* = .869; −0.8%, *p* = .952, respectively) and significantly increased in the FOS group (613.0%, *p* < .001; 69.9%, *p* < .001, respectively). At the individual level, all subjects in the FOS group increased their O3i.

### Dietary intake

All dietary data, normalized by body mass (kg) for the macronutrients, are reported in Table 2. In brief, there were no significant differences between groups nor over time in self-reported calorie and macronutrient intake. Dietary intake of EPA and DHA was similar between groups and did not change from PRE to POST.

**Table 2.** Nutritional analysis.

	Group	Pre, Mean ± SD	Post, Mean ± SD	*p* (Time)	*p* (GxT)
Total energy (kcal)	FO	1889.2 ± 343.0	1905.1 ± 462.7	.676	.994
	PL	1901.1 ± 520.6	1916.5 ± 414.7		
Carbohydrate (g∙kg^−1^)	FO	2.6 ± 1.0	2.6 ± 1.0	.985	.868
	PL	2.7 ± 1.4	2.7 ± 0.9		
Protein (g∙kg^−1^)	FO	1.5 ± 0.3	1.5 ± 0.2	.889	.690
	PL	1.2 ± 0.3	1.3 ± 0.2		
Fat (g∙kg^−1^)	FO	1.0 ± 0.2	1.0 ± 0.3	.529	.938
	PL	1.0 ± 0.4	0.9 ± 0.2		
EPA (mg)	FO	29.6 ± 28.8	17.6 ± 18.6	.306	.114
	PL	13.9 ± 22.4	16.6 ± 17.1		
DHA (mg)	FO	63.8 ± 59.1	39.6 ± 41.0	.345	.104
	PL	33.3 ± 53.0	40.0 ± 37.8		

Amount of EPA and DHA does not include supplementation

Abbreviations: FO, fish oil; PL, placebo; EPA, eicosapentaenoic acid; DHA, docosahexaenoic acid

### Volume load

**Figure 4.**
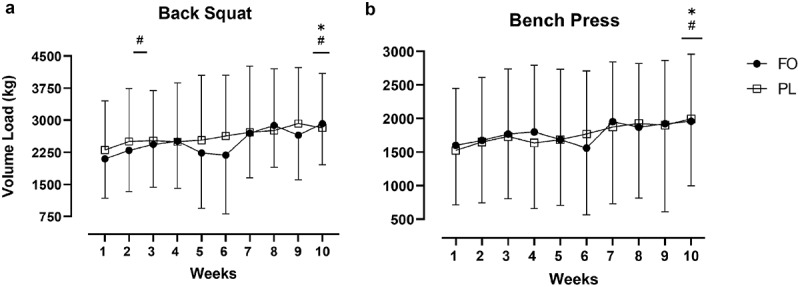
Weekly Volume Load for the a) Back Squat and b) Bench Press. Data are mean ± SD.*significantly different than week 1 for PL (Back Squat: *p* = .016; Bench Press: *p* = .001), #significantly different than week 1 for FO (Back Squat: week 3, *p* = .003; week 10, *p* < .001; Bench Press: *p* = .031). There were no group by time interactions (*p* > .05).

Total volume load over the 10 weeks was similar between conditions (FOS: 42,670 ± 18,925 kg, PL: 43,879 ± 22,765 kg, *p* = .897). There were no between-group differences in total volume load for the back squat (FOS: 24,888 ± 9,985 kg, PL: 26,197 ± 12,914 kg, *F* (1,19) = 0.017, *p* = .897) or bench press (FOS: 17,782 ± 9,173 kg, PL: 17,683 ± 10,033 kg, *p* = .897). Back squat and bench press volume load significantly increased over 10-weeks (*p*< .001). Compared to baseline, back squat volume load was significantly higher at week 3 (*p* < .001), week 8 (*p* = .016), week 9 (*p* = .006), and week 10 (*p* < .001). For bench press, volume load was significantly higher in week 3 (*p* = .025), week 8 (*p* = .049), and week 10 (*p* < .001) compared to week 1. Volume load data for the back squat and bench press over 10-weeks and between groups are noted in Figure 4.

### Body composition

As indicated in Table 3, there was no significant between-group differences in LBM (FOS: +3.4%, PL: +2.4%), FM (FOS: −5.2%, PL: 0.0%), nor %BF (FOS: −5.9%, PL: −2.5%). Notably, the between-group magnitude was considered moderate and large for %BF (−0.91, 95%CI: −2.13, 0.31, *d* = 0.74) and FM (−1.08 kg, 95%CI: −2.36, 0.20, *d* = 0.84), respectively, favoring the FOS group. The 0.6 kg difference in LBM, favoring the FOS group, was considered small (*d* = 0.36).

**Table 3.** Data of main study outcomes.

Outcomes	Group	Pre, Mean ± SD	Post, Mean ± SD	Unadjusted ∆ ± SD	Baseline Adjusted ∆ (CI)	*p* (Group)	ES
Squat 1RM (kg)	FO	82.9 ± 35.0	106.8 ± 38.4	23.9 ± 8.1	24.2 (17.5, 30.9)	.191	0.64
	PL	90.9 ± 43.1	109.5 ± 48.6	18.6 ± 12.1	18.2 (11.8, 24.6)		
Bench 1RM (kg)	FO	62.7 ± 37.0	73.9 ± 40.7	11.1 ± 6.8	11.3 (7.7, 14.8)	.**047**	**1.00**
	PL	66.3 ± 37.0	72.7 ± 39.6	6.4 ± 5.0	6.3 (2.9, 9.7)		
LBM (kg)	FO	55.9 ± 15.6	57.9 ± 16.7	1.9 ± 1.9	2.0 (0.8, 3.1)	.457	0.36
	PL	58.2 ± 14.9	59.6 ± 15.4	1.4 ± 1.7	1.4 (0.3, 2.5)		
FM (kg)	FO	17.4 ± 3.8	16.5 ± 3.1	−0.91 ± 1.2	−1.0 (−1.9, −0.1)	.092	**0.84**
	PL	33.3 ± 53.0	19.3 ± 5.6	−0.02 ± 1.6	0.1 (−0.8, 0.9)		
BF (%)	FO	23.9 ± 6.9	22.4 ± 6.2	−1.4 ± 1.5	−1.5 (−2.4, −0.6)	.136	0.74
	PL	24.9 ± 8.0	24.2 ± 7.3	−0.63 ± 1.6	−0.6 (−1.4, 0.3)		

**Bold** indicates a significant *p*-value (p < .05) or large effect size (≥ 0.8)

Abbreviations: **∆**, change; CI, 95% confidence interval (upper bound, lower bound); ES, effect size (Cohen’s *d*); 1RM, 1-repetition maximum; LBM, lean body mass; FM, fat mass; BF, body fat

### Strength testing

**Figure 5.**
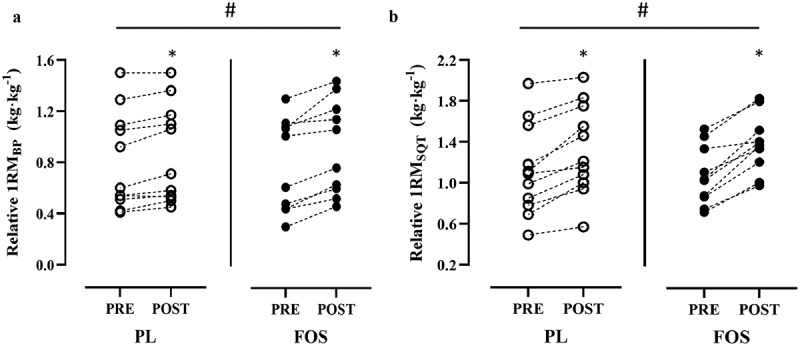
Individual Data Points of the Change in Relative a) 1RM Bench Press (1RM_BP_) and b) Back Squat (1RM_SQT_) Before (PRE) and After (POST) 10-Weeks of Resistance Exercise Training and Supplementation (placebo [PL, *n* = 11] or fish oil [FOS, *n* = 10])*significant change from PRE for relative 1RM_BP_ (PL, *p* = .006; FOS, *p* < .001) and 1RM_SQT_ (PL, *p* < .001; FOS, *p* < .001); #significant difference between groups for relative1RM_BP_ (PL: 7.3% vs. FOS: 17.6%, *p* = .011) and 1RM_SQT_ (PL: 17.9% vs. FOS: 29.3%, *p* = .045).

The 1RM strength data are shown in Table 3. Relative to PL, 10-weeks of FOS and RET increased absolute 1RM_BP_ (5.0 kg, 95%CI: 0.07, 9.93, *p* = .047) and tended to increase absolute 1RM_SQT_ (6.0 kg, 95%CI: −3.29, 15.31, *p* = .191). Figure 5 depicts the change in relative 1RM strength from PRE to POST. Briefly, the change in relative 1RM_BP_ and 1RM_SQT_ was significantly higher in the FOS group compared to PL (0.14 kg∙kg^−1^ vs. 0.06 kg∙kg^−1^, *p* = .011 and 0.31 kg∙kg^−1^ vs. 0.20 kg∙kg^−1^, *p* = 0.045, respectively).

## 4. Discussion

To our knowledge, this is the first study to investigate the effects of FOS compared to PL on skeletal muscle adaptations following a 10-week RET program in young adults, while using a membrane-centric hypothesis. We demonstrated that 3.85 g combined EPA and DHA daily for 10-weeks resulted in significantly greater RET-induced gains in absolute and relative 1RM_BP_ and relative 1RM_SQT_. Although we found moderate-to-large between-group differences based on ES for absolute 1RM_SQT_, %BF, and FM, they were statistically equivocal to PL. Contrary to our hypothesis, FOS failed to differentially influence LBM compared to PL.

In young adults, changes in RET-induced muscular strength are often associated with changes in muscle mass [2]. While LBM increased in both groups within the present investigation, 1RM strength – especially relative strength (kg∙kg^−1^ body mass) – improved to a greater extent in the FOS group compared to PL. Given that MPS is the primary contributor to hypertrophy in young adults [2,34], we hypothesized that strength improvements with FOS would be mediated by concomitant LBM enhancements. However, despite demonstrating similar between-group LBM changes, absolute 1RM_BP_, relative 1RM_BP_, and relative 1RM_SQT_ were improved by 4.7 kg (8%), 0.08 kg∙kg^−1^ (10.3%), and 0.11 kg∙kg^−1^ (11.4%), respectively, more than PL. While the relationship between muscle mass and strength is well-established, muscle quality, or strength per unit of muscle mass, can improve through several factors both independently or commensurate to LBM [35]. Thus, beyond hypertrophy, other factors such as fiber type distribution, reductions in intramuscular fat, and enhanced neuromuscular activation may have uniquely improved measures of muscular strength [36].

Although speculative, the baseline adjusted between-group LBM difference of 0.6 kg (+1.2%, *p* > .05), may be partially explained by enhanced MPS. FOS in the presence of nutrient stimuli has been shown to improve MPS by 50% in young adults [7]. However, this effect appears to be modestly attenuated following an acute bout of RET in a similar demographic [8]. Notwithstanding that trained-individuals experience a somewhat blunted RET-mediated MPS response [37], we expected a more pronounced MPS-associated LBM accrual among our recreationally trained subjects. Nevertheless, MPS changes following an acute RET bout may not correlate with chronic RET-induced skeletal muscle hypertrophy [38,39]. The between-group differences observed in the present investigation were nonetheless statistically equivocal; regardless, it remains plausible that FOS and its subsequent skeletal muscle phospholipid incorporation upregulated muscle protein synthetic machinery, albeit to a much smaller degree than expected. Evidence suggests that the efficacy of omega-3 fatty acids on the activity of anabolic cell signaling pathways and, thus, LBM may be acutely influenced by protein intake. While previous studies have reported substantial increases in MPS (~50-100%) with FOS [6,7,40], McGlory et al. [8] recently reported that MPS may not be acutely upregulated by omega-3 fatty acids in the presence of optimal protein intake. Since our participants reported dietary protein intakes ≥ 1.2 g∙kg^−1^, it is possible that the muscle protein synthetic machinery was saturated to the extent that FOS would not have exerted an additional anabolic response and, by extension, similar rates of LBM accrual.

Other alternate explanations for the FOS-mediated greater magnitudes of 1RM strength improvement despite similar LBM augmentations may be related to muscle quality and its associated factors such as muscle fiber type transition and neuromuscular function. Since relative strength in the bench press and back squat changed to a greater degree than absolute strength compared to PL, FOS most likely influenced mechanisms related to muscle quality. Although performed in elderly populations, many fish oil supplementation studies with concurrent RET have noted similar strength improvements without significant LBM changes [11,24]. Furthermore, a study in resistance-trained young men found that 4 g∙d^−1^ FOS improved 1RM leg extension, notably despite a loss in muscle mass during a 40% calorie restricted diet [41]. While our study protocol led to hypertrophy in both groups, this illustrates the ability of fish oil supplementation to improve strength even in an otherwise compromised physiological environment. This may partially be explained by fast-twitch muscle fiber hypertrophy [42]. In older adults, 6 weeks of 3.68 g∙d^−1^ LC n-3 PUFA (1.86 g EPA, 1.54 g DHA) administration alongside RET significantly increased fast-twitch muscle fiber cross-sectional area (fCSA) in the absence of whole-body LBM alterations [42]. These data corroborated similar investigations’ fiber type-specific data following LC n-3 PUFA supplementation [43,44]. While LBM increased to a similar degree between groups, it is plausible, although highly speculative, based on our differential 1RM strength outcomes, that fCSA increased to a greater extent in the FOS group. Our FOS protocol was intentionally EPA-biased (1.4:1, EPA:DHA) since it has been shown to uniquely influence MPS [12]; however, DHA is the prominent fatty acid involved in neuromuscular control [45]. As previously hypothesized by Philpott et al. [41], the DHA component of our supplement significantly increased blood DHA (~70%), thus, it is reasonable to assume that neural DHA concomitantly increased. Consequently, Rodacki et al. [13] demonstrated greater neural activation (i.e. faster muscular response after a stimulus) following 90 days of combined FOS and RET. Previous authors have reported that neuromuscular adaptations may occur as early as 21-days following FOS, although the effect may be attenuated compared to studies using higher doses or longer durations [46]. In agreement with previous investigations [41], these data may therein support a FOS-mediated neuromuscular enhancement that ultimately influenced the observed strength outcomes.

Much of the evidence to date on RET and FOS is in older adults and largely reports favorable results for LC n-3 PUFA supplementation [11,13,24,42]. To our knowledge, there are only two studies that used a RET protocol combined with n-3 PUFA supplementation alone or as part of a protein-based supplement in young adults [18,19]. Specifically, Georges et al. [18] found that 8-weeks of RET with 3 g·d^−1^ krill oil administration significantly improved both LBM and 1RM bench and leg press from PRE to POST in young trained men; however, the results were nonetheless similar to placebo. Hayward et al. [19] presented similar findings in untrained females amidst a combined 4-week RET program and protein-based supplement containing n-3 PUFAs. Although LBM and strength were improved from baseline, no significant differences were noted compared to controls. The present study also reported similar changes in LBM compared to PL; however, our investigation was the first to report greater improvements in strength outcomes. Notably, our observed FOS-mediated strength increases compared to other studies are ostensibly due to the skeletal muscle LC n-3 PUFA incorporation, likely facilitated by our more optimal dosing regimen. In line with our results, a recent systematic review and meta-analysis, albeit in older adults, found that FOS does not increase LBM; however, it does improve strength with or without RET [47].

Although there are significant strengths to the present investigation, such as the inclusion of young females to further our understanding of the effect of FOS and RET across the general young adult population, blood LC n-3 PUFA status measurement, equivalent macronutrient intake, as well as similar volume loads and RET-induced strength changes compared to resistance trained subjects [27,28,48], this study was not without limitations. While the number of subjects enrolled in our study was similar to previous investigations, it’s possible that our study may have been underpowered to detect significant between-group changes in certain outcomes, especially in light of our wide confidence intervals on some measures (Table 3). The DXA is widely considered the reference method to determine changes in body composition when using a standardized protocol; however, our findings could have been strengthened with the use of total body water or more direct methods of site-specific skeletal muscle mass and type quantification (MRI, muscle biopsies, etc.). Lastly, subject diets were not controlled and, although unlikely, it remains possible that dietary fluctuations may have unintentionally influenced the participants’ PRE-to-POST body composition.

## 5. Conclusion

In summary, our data confirms, once again, that RET is key for beneficial skeletal muscle adaptations and body recomposition in young healthy men and women. The addition of fish oil supplementation (4 g∙d^−1^ [3.85 g EPA+DHA]) to a 10-week RET program may augment absolute 1RM_BP_ and relative 1RM_BP_ and 1RM_SQT_, and a greater reduction in FM. It is unclear if FOS increases LBM to a greater extent than RET alone. In light of our results and previous findings regarding the efficacy of FOS in young and athletic populations [14,49], FOS may be explored as a feasible and cost-effective nutritional strategy to influence general health and training adaptations, primarily for those with low blood or suboptimal dietary intake of LC n-3 PUFAs. Unlike targeted pharmaceutical interventions, the complex and often unspecified action of LC n-3 PUFAs – especially the notable divergent actions of EPA and DHA – on human physiology can make identification of an underlying mechanism challenging. Nevertheless, the convergence of multiple known contributors, including MPS, muscle quality characteristics, and neuromuscular control, likely contributed to our findings. As Anthony and colleagues [16] antecedently suggest, future research in healthy, young trained personnel is warranted to examine the influence of FOS on RET-induced adaptations using more precise tracer, muscle biopsy, and neuromuscular assessments to ascertain the aforementioned underlying mechanisms. Additionally, these subsequent investigations should explore the differential effects of EPA and DHA on skeletal muscle functional outcomes, potentially benefiting from longer duration (>10 weeks) supplementation protocols and doses equivalent to 3 servings of fatty fish per week.
